# A 2 year retrospective study of vegan patients and their pregnancy outcomes in a tertiary level Irish hospital

**DOI:** 10.1002/ijgo.71055

**Published:** 2026-04-30

**Authors:** Mona Abdelrahman, Siofra Maher, Stephen W. Lindow, Michael P. O'Connell

**Affiliations:** ^1^ The Coombe Hospital Dublin Ireland

**Keywords:** diet, maternal health, perinatal health, pregnancy, vegan

## Abstract

**Objective:**

Recent previous studies have revealed outcomes of higher risk of low birth weight, small for gestational age, and pre‐eclampsia for vegan pregnant women compared to other diets. This review examined if patients with vegan diet in our tertiary hospital had similar outcomes.

**Methods:**

A retrospective cohort study was conducted for 2022–2024 examining the factors of those who ate an unrestricted diet versus vegan diet (10 270 vs 68 women). Data included maternal demographics, clinical factors and pregnancy outcomes. Statistical analysis was performed using SPSS version 29.

**Results:**

Vegans demonstrated a lower body mass index (BMI) than those with an unrestricted diet (*P* < 0.05). There was no difference in reported anemia in vegans versus those with unrestricted diets (17.6% vs 16.1%, *P* = 0.73) and no significant risk for requiring a blood transfusion either (2.9% vs 3.4%, *P* = 0.84), respectively. There was no statistically significant difference between the outcomes of those with a vegan versus unrestricted diet in median birth weight (3390 vs 3410 g, *P* = 0.77) or rate of premature delivery <37 weeks (7.4% vs 9.5%, *P* = 0.55). There was also no statistically significant difference for developing gestational diabetes (8.8% vs 9.9%, *P* = 0.77), or gestational hypertension (0% vs 3.5%, *P* = 0.12) for vegans versus unrestricted diets, respectively.

**Conclusion:**

The outcome in pregnancy for women with vegan and unrestricted diets was equivalent in our cohort. There is limited research on the consequences of vegan diets in pregnancy and further observational longitudinal studies are required for more robust data. Socioeconomic factors should be taken into consideration.

## 1 INTRODUCTION

### 1.1 Background

Pregnancy is a critical period that demands optimal nutrition to support both maternal health and fetal development. With the rising popularity of plant‐based diets, including veganism, questions have arisen regarding the adequacy of such diets during pregnancy. Veganism, defined as the practice of not eating or using any animal products,^1^ has been increasing in recent years in response to the ongoing climate crisis and new data elucidating the potential health benefits of this diet. UK government figures suggest approximately 3% of the population following a vegan diet. This is an increase from their initial report in 2019.^2^ It is also in line with American data which estimates 6% of the population following a vegan diet.^3^, ^4^ Recent studies from Segovia‐Siapco et al., and the European Prospective Investigation into Cancer and Nutrition showed both vegetarian and vegan diets of equicaloric equivalent to carnivore diets had a 29% and 47% reduction to greenhouse gas emissions, respectively.^5^

Pregnancy is a time when a well balanced nutrient dense diet is needed. Professional bodies, including the Academy of Nutrition and Dietetics, have stated that well‐planned vegan diets are appropriate during all life stages, including pregnancy.^6^ Documentation of improved health outcomes such as lower rates of type 2 diabetes, coronary artery disease and cancer have been linked to a vegan diet. Conversely, a strict vegan diet has also been showed to increase risk of micro‐ and macronutrient deficiency.^7^, ^8^, ^9^ Pregnancy is a time in which nutrition is of upmost importance and increase in micronutrients is seen. Key nutrients of concern in a vegan during pregnancy include vitamin B12, iron, calcium, vitamin D, iodine, omega‐3 fatty acids, and protein. Deficiencies in these can increase the risk of complications such as low birth weight,^10^, ^11^ neural tube defects, and impaired neurodevelopment.^12^, ^13^ However, with careful dietary planning and appropriate supplementation, these risks can be minimized.

There has been a trend in vegetarian and vegan diets worldwide and this may be due to efforts in sustainable practices with the ongoing climate crisis. Segovia‐Siapco and Sabate noted that greenhouse gas emissions of equicaloric diets are 29% less in vegetarian diet in the Adventist Health Study‐2 (AHS‐2) and 47%–60% less for vegetarian/vegan diets in European Prospective Investigation into Cancer and Nutrition‐Oxford (EPIC‐Oxford) than non‐vegetarian/meat‐eating diets. Vegans in AHS‐2 have 16% reduced risk while vegans, vegetarians, and fish‐eaters in EPIC‐Oxford have 11%–19% lower risk for all cancers compared to non‐vegetarians.^5^ Adopting vegetarian diets addresses health benefits while reducing carbon footprint.

In terms of nutritional effects of diets, Bakaloudi et al. performed a systematic review that showed following a vegan diet may result in deficiencies in protein intake and micronutrients (vitamin B12, zinc, calcium and selenium). Interestingly, the review also revealed that iron intake seemed higher for those that followed a vegan diet compared to other diets as they tend to consume more green leafy vegetables, grains, nuts and beans which are iron‐rich foods but overall, low ferritin concentrations are observed in vegans.^14^ This may be attributed to the lower bioavailability of iron in plant based food when digested.

### 1.2 Objectives

There have been previous studies examining the outcomes in pregnancies for mothers that adhere to a vegan diet. These studies revealed outcomes of low birth weight, higher risk of small for gestational age, and risks of pre‐eclampsia compared to other diets.^15^, ^16^ The present study set out to examine if a vegan diet in a tertiary maternity hospital in Ireland has similar birth outcomes to enable clinicians to further counsel women.

## 2 MATERIALS AND METHODS

The Coombe Hospital keeps a contemporaneous computer‐based record of all women who book for antenatal care following a detailed history with the midwife and informed consent. This record is updated after delivery with all maternal, neonatal and delivery outcomes details. Since June 2022, the Coombe hospital has been recording data about the women's diet in pregnancy at their first visit, with their consent, with the options including unrestricted diet, vegan, vegetarian or pescatarian. Patients were asked about their dietary preferences by a midwife and had the opportunity to ask questions/clarifications. A vegan diet is defined as a diet that does not include any dairy, eggs, or products from any animal sources.

A retrospective analysis of this database between June 2022 and July 2024 of women who ate an unrestricted diet and a vegan diet was retrieved. Data including type of diet, maternal demographic data, clinical factors, history of anemia, pregnancy complications, pregnancy outcomes, and birth weights were analyzed, and all data was anonymized. A total of 10 270 women who ate an unrestricted diet and 68 women who ate a vegan diet were examined from their first visit appointment to their delivery. Statistical analysis with the Mann–Whitney *U* (MWU) and chi‐square tests was performed using SPSS Statistics for Windows 29.0 (IBM, Armonk, New York, USA).

The study was approved by the Audit and Quality Advisory group (approval reference no. 24‐07‐01) at the Coombe Hospital, which authorized the audit of hospital data.

## 3 RESULTS

Overall, 10 270 women who ate an unrestricted diet and 68 women who ate a vegan diet were analyzed. The results for maternal age, body mass index (BMI calculated as weight in kilograms divided by the square of height in meters), last recorded hemoglobin, birth weight and gestation at delivery were found not to be normally distributed (Kolmogorov–Smirnov statistic 0.071, 0.086, 0.028, 0.082, and 0.223 respectively; all *P* < 0.001). Therefore, non‐parametric tests were used for analysis.

Table 1 outlines the baseline maternal characteristics. Notably, the vegan women demonstrated a significantly lower BMI than the women who followed an unrestricted diet (*P* < 0.05). Vegan women were more likely to be of Caucasian versus South Asian origin (72.1% vs 23.5%, respectively) and have a blood relative as the baby's father (5.9% vs 1.4% of vegan vs unrestricted diets). There was no evidence of an increased rate of antenatal anemia in vegan women, 17.6% reported anemia prior to pregnancy, versus 16.1% of those with an unrestricted diet (*P* = 0.73).

**TABLE 1:** Baseline maternal characteristics for women with an unrestricted versus a vegan diet in pregnancy.

Maternal characteristics	Unrestricted diet *N* = 10 270	Vegan diet *N* = 68	Statistical significance
Median (range) maternal age at delivery (years)	33 (14–54)	32 (21–43)	MWU –0.97 *P* = 0.33 (NS)
Median (range) BMI	25.8 (10.4–82.6)	24.3 (17–35.9)	MWU –2.1 *P* < 0.05
Primiparous *N* (%)	4457 (43.4%)	32 (47.1%)	Chi‐square 0.37 *P* = 0.54 (NS) OR 1.16 (0.72–1.9)
Primigravida *N* (%)	3305 (32.2%)	25 (36.8%)	Chi‐square 0.65 *P* = 0.42 (NS) OR 1.2 (0.75–2.0)
Ethnicity Caucasian *n* (%)	8586 (83.6%)	49 (72.1%)	Chi‐square 0.6 *P* = 0.4 (NS) OR 0.8 (0.47–1.37)
Ethnicity South Asian *n* (%) *India/Pakistan/Bangladesh	722 (7.0%)	16 (23.5%)	Chi‐square 27.7 *P* < 0.001 OR 4.1 (2.3–7.2)
Member of traveling community *n* (%)	226 (2.2%)	3 (4.4%)	Chi‐square 1.5 *P* = 0.22 (NS) OR 0.49 (0.16–1.6)
No medical problems in pregnancy *n* (%)	7618 (74.2%)	51 (75%)	Chi‐square 0.05 *P* = 0.8 (NS) OR 1.0 (0.6–1.8)
Median (range) last antenatal hemoglobin measurement	11.8 (6.0–16.6)	12.1 (9.6–14.5)	MWU 1.5 *P* = 0.14 (NS)
Alcohol currently taken *N* (%)	36 (0.35%)	0	Chi‐square 0.24 *P* = 0.6 (NS) OR 2.1 (0.1–33.7)
Previous use of illicit drugs *N* (%)	665 (6.5%)	4 (5.9%)	Chi‐square 0.04 *P* = 0.8 (NS) OR 0.90 (0.3–2.5)
Ever smoked *n* (%)	3332 (32.4%)	17 (25%)	Chi‐square 1.7 *P* = 0.19 (NS) OR 0.69 (0.4–1.2)
Smoke currently *N* (%)	687 (6.7%)	1 (1.5%)	Chi‐square 3.0 *P* = 0.09 (NS) OR 0.21 (0.03–1.5)
Previous history of anemia in pregnancy *n* (%)	1019 (9.9%)	5 (7.4%)	Chi‐square 0.5 *P* = 0.48 (NS) OR 0.72 (0.3–1.8)
Previous history of anemia outside of pregnancy *n* (%)	1652 (16.1%)	12 (17.6%)	Chi‐square 0.12 *P* = 0.73 (NS) OR 1.1 (0.6–2.1)
Previous anemia treatment (including dietary advice) *n* (%)	3492 (34%)	24 (35.3%)	Chi‐square 0.05 *P* = 0.8 (NS) OR 0.95 (0.6–1.6)
Previous blood transfusion *n* (%)	454 (4.4%)	3 (4.4%)	Chi‐square 1.3 *P* = 0.94 (NS) OR 1.0 (0.3–3.2)
Diabetes developed in pregnancy	1016 (9.9%)	6 (8.8%)	Chi‐square 0.08 *P* = 0.77 (NS) OR 0.9 (0.4–2.0)
Treatment for raised blood pressure in pregnancy *n* (%)	356 (3.5%)	0	Chi‐square 2.4 *P* = 0.12 OR 0.2 (0.1–3.3)
Father of baby is a blood relative *n* (%)	147 (1.4%)	4 (5.9%)	Chi‐square 9.3 *P* < 0.01 OR 4.3 (1.6–12.0)

*Note*: BMI, calculated as weight in kilograms divided by the square of height in meters.

Abbreviations: BMI, body mass index; MWU, Mann–Whitney U test; NS, not significant; OR, odds ratio.

Table 2 demonstrates the pregnancy outcomes. Notably, there was no statistically significant difference between the outcomes of those with an unrestricted versus a vegan diet in the median birth weight (3410 vs 3390 g) or the rate of premature delivery <37 weeks (9.5% vs 7.35%, respectively). Of note, there was also no statistically significant difference in the two groups of patients for developing gestational diabetes (9.9% vs 8.8%, *P* = 0.77), or gestational hypertension (3.5% vs 0%, *P* = 0.12) for an unrestricted versus a vegan diet, respectively.

**TABLE 2:** Pregnancy outcomes for women with an unrestricted versus a vegan diet.

Pregnancy outcomes	Unrestricted diet *N* = 10 270	Vegan diet *N* = 68	Statistical significance
Median (range) birth weight (g)	3410 (242–5740)	3390 (1380–4270)	MWU –0.29 *P* = 0.77 (NS)
Median (range) total gestation (weeks)	39 (22–44)	39 (30–41)	MWU 0.58 *P* = 0.56 (NS)
Cesarean section *N* (%)	4011 (39.1%)	33 (48.5%)	Chi‐square 2.5 *P* = 0.11 (NS) OR 1.5 (0.9–2.4)
Gestation *N* (%)			Chi‐square 3.4 *P* = 0.34 (NS)
22–27 weeks	118 (1.1%)	0	
28–33 weeks	229 (2.2%)	3 (4.4)	
34–36 weeks	629 (6.1%)	2 (2.9)	
37–45 weeks	9294 (90.5%)	63 (92.6%)	
Total number of premature births *N* (%)	976 (9.5%)	5 (7.4%)	Chi‐square 0.36 *P* = 0.55 (NS) OR 0.76 (0.31–1.9)
Stillbirth *n* (%)	43 (0.4%)	0	Chi‐square 0.29 *P* = 0.59 OR 0.58 (0.04–9.6)
Apgar at 1 min <7	595 (5.8%)	3 (4.5%)	Chi‐square 0.19 *P* = 0.66 (NS) OR 0.77 (0.24–2.5)
Apgar at 5 min <7	122 (1.2%)	1 (1.5%)	Chi‐square 0.06 *P* = 0.81 (NS) OR 1.3 (0.18–9.1)
Normal initial neonatal examination *n* (%)	9870 (96.1%)	66 (97.1%)	Chi‐square 0.16 *P* = 0.69 (NS) OR 1.3 (0.3–5.5)
Blood loss at birth <500 mL *N* (%)	7361 (71.7%)	48 (70.6%)	Chi‐square 0.04 *P* = 0.84 OR 0.95 (0.6–1.6)
Admitted to maternal HDU	231 (2.2%)	0	Chi‐square 1.6 *P* = 0.21 (NS) OR 0.31 (0.02–5.1)
Blood transfusion *N* (%)	349 (3.4%)	2 (2.9%)	Chi‐square 0.04 *P* = 0.84 (NS) OR 0.9 (0.2–3.5)

Abbreviations: HDU, high dependency unit; MWU, Mann–Whitney *U* test; NS, not significant; OR, odds ratio.

In summary, the outcome in pregnancy for vegan women was equivalent to the outcome in women who ate an unrestricted diet in our cohort of patients.

## 4 DISCUSSION

A growing body of research has examined how vegan diets during pregnancy compare to omnivorous (non‐vegan) diets in terms of maternal health, fetal development, and birth outcomes. In this study, we examined the association of various fetal outcomes, maternal outcomes and maternal characteristics with a vegan and non‐vegan diet. We assessed parameters such as fetal birth weight, maternal weight, neonatal admission to the intensive care unit, and maternal need for blood transfusion. None of the examined birth outcomes was statistically significant between groups. Maternal characteristics were also examined, and significant findings are in keeping with the literature. One limitation of our study is that during the 2‐year interval only a small minority of women booked for antenatal care were observing a vegan diet, and therefore our data cannot be generalized to all women with a vegan diet.

The data from this study does not demonstrate a statistically significant lower birth weight between groups. This differs from recent literature from Hedegarrd et al., who examined more than 60 000 pregnancies between 1996 and 2002, and revealed that patients who had a vegan diet gave birth to babies with lower mean birth weights and had higher rates of pre‐eclampsia compared to omnivore patients.^15^ Avnon et al. also compared vegan diet outcomes in pregnancy with omnivorous, pescatarian and lacto‐ovo‐vegetarians. They found that a vegan diet was associated with an increased risk of small for gestational age births and lower birth weights in addition.^16^ Kesary et al. published data examining diet in pregnancy in approximately 1500 women. In their study, an association of maternal diet during pregnancy and certain perinatal outcomes were found. In this cohort, a vegan diet during pregnancy was associated with a reduced risk of excessive maternal weight gain and also an increased risk of small for gestational weight and lower birth weight centile.^17^ However, a systematic review published by Sebastiani et al. found no significant differences in birth weight between vegan/vegetarian and omnivorous mothers,^10^ although some of the aforementioned studies suggested slightly lower average birth weights in plant‐based groups, possibly due to lower gestational weight gain.

Plant‐based diets, such as a vegan diet, may reduce the risk of gestational diabetes mellitus (GDM) by improving insulin sensitivity, attributable to low glycemic load and high dietary fiber. A large cohort study in China indicated a reduced risk of GDM in women adhering to plant‐based diets^18^; however, the effect among strict vegans requires further investigation. In our data no significant difference between rates of GDM in the vegan and non‐vegan group was seen.

Our data did not note a difference in pre‐term birth rates in vegan versus non‐vegan women. Although there is no clear association between preterm delivery and type of diet, some studies have revealed a higher risk of preterm delivery rate with higher consumption of animal and dairy products^19^, ^20^ as well as artificially sweetened beverages.^21^, ^22^ A Norwegian study also revealed a lower risk of preterm delivery with an increased vegetable/fruit/whole grain‐type diet compared to “western” diets of processed snacks and meat products.^23^

In a systematic review of dietary patterns and risk of hypertensive disorders in pregnancy (HDP), diets characterized by a high intake of vegetables, fruits, whole grains, nuts, legumes, fish and vegetable oils were associated with a 30%–42% decreased risk of HDP and a 14%–29% decreased risk of pre‐eclampsia.^24^ This is also in keeping with a more recent study in 2024 that evaluated two diet types and revealed a lower risk of HDP in those who consumed more vegetables and fruits compared to dairy and refined grains.^25^ Specific data on vegan pregnancies remains sparse. In the data from this research study, no difference was seen between vegan and non‐vegan groups in terms of hypertensive disorders in pregnancy.

Sociodemographic profiles of meat‐eaters, vegetarians and vegans were compared, and it was found that vegetarians were more likely to be females and have a higher educational level, whereas vegans had a lower education level.^26^ This suggests that the type of diet may be based on financial means rather than sustainability or health choices. The same study was conducted via a self‐administered questionnaire so gender and educational level are suggestive of confounding factors.^27^

## 5 CONCLUSION

In conclusion, a healthy vegan during pregnancy requires diligent planning and attention to nutrient intake and potentially nutritional supplementation. By working with healthcare professionals, focusing on a diverse diet, and considering supplementation, vegans should achieve a healthy and successful pregnancy but there is limited research on the consequences of vegan diets in pregnancy. More observational longitudinal studies are required for further robust data to ascertain the association between diet and pregnancy outcomes and demographic factors such as ethnicity and socioeconomic class should be taken into consideration.

## AUTHOR CONTRIBUTIONS

The conceptualization of the study was by M. Abdelrahman and S. W. Lindow. Methodology included data curation and formal analysis by S. W. Lindow. Writing of original draft and research of references by M. Abdelrahman. Edits and review were provided by S. Maher and S. W. Lindow. All authors have read, critically reviewed, edited and agreed to the published version of the manuscript. Supervision by S. W. Lindow and M. P. O'Connell.

## FUNDING INFORMATION

The authors have nothing to report.

## CONFLICT OF INTEREST STATEMENT

The authors have no conflicts of interest.

## Data Availability

Data sharing is not applicable to this article as no new data were created or analyzed in this study.
